# Cell Surface Fibroblast Activation Protein-2 (Fap2) of *Fusobacterium nucleatum* as a Vaccine Candidate for Therapeutic Intervention of Human Colorectal Cancer: An Immunoinformatics Approach

**DOI:** 10.3390/vaccines11030525

**Published:** 2023-02-23

**Authors:** Somrita Padma, Ritwik Patra, Parth Sarthi Sen Gupta, Saroj Kumar Panda, Malay Kumar Rana, Suprabhat Mukherjee

**Affiliations:** 1 Integrative Biochemistry & Immunology Laboratory, Department of Animal Science, Kazi Nazrul University, Asansol 713340, West Bengal, India; 2 School of Biosciences & Bioengineering, D. Y. Patil International University, Akurdi, Pune 411044, Maharashtra, India; 3 Department of Chemistry, Indian Institute of Science Education and Research, Berhampur 761008, Odisha, India

**Keywords:** human colorectal cancer, *Fusobacterium nucleatum*, fibroblast activation protein-2 (Fap2), toll-like receptors (TLRs), multi-epitope peptide vaccine, cloning, molecular docking, immune simulation

## Abstract

Colorectal cancer (CRC) is one of the most common cancers and is the second-highest in cancer-related deaths worldwide. The changes in gut homeostasis and microbial dysbiosis lead to the initiation of the tumorigenesis process. Several pathogenic gram-negative bacteria including *Fusobacterium nucleatum* are the principal contributors to the induction and pathogenesis of CRC. Thus, inhibiting the growth and survival of these pathogens can be a useful intervention strategy. Fibroblast activation protein-2 (Fap2) is an essential membrane protein of *F. nucleatum* that promotes the adherence of the bacterium to the colon cells, recruitment of immune cells, and induction of tumorigenesis. The present study depicts the design of an in silico vaccine candidate comprising the B-cell and T-cell epitopes of Fap2 for improving cell-mediated and humoral immune responses against CRC. Notably, this vaccine participates in significant protein–protein interactions with human Toll-like receptors, especially with TLR6 reveals, which is most likely to be correlated with its efficacy in eliciting potential immune responses. The immunogenic trait of the designed vaccine was verified by immune simulation approach. The cDNA of the vaccine construct was cloned in silico within the expression vector pET30ax for protein expression. Collectively, the proposed vaccine construct may serve as a promising therapeutic in intervening *F. nucleatum*-induced human CRC.

## 1. Introduction

Colorectal cancer (CRC) is one of the most common forms of human cancer and is currently ranked in second position among all cancer-related deaths worldwide [1]. In the year 2020, a global study conducted on the prevalence of CRC demonstrated the emergence of 1.93 million new cases of CRC and around 0.93 million cancer-related deaths [2]. The occurrence of new cases was found to be highest in the United States and China. The epidemiological status of CRC in India stated the occurrence of 7.2 per lakh male population and 5.1 per lakh female population [3]. The actual mechanism of the malignancy of CRC is still not well understood. However, it is known that the progression of colon carcinoma is a multi-factorial and multi-step process [1]. It begins with the initiation of chronic inflammation in a healthy colon followed by initial low-grade to final high-grade dysplasia [1,4].

The human gut microbiota contains millions of bacteria, archaea, fungi, protozoans, and viruses [5]. These microorganisms play a vital role in various physiological processes including digestion, metabolism, epithelial homeostasis, and gut lymphoid tissue development, as well as maintaining the physiological and immune homeostasis of the gut and extra-gut organs/tissues [5,6,7]. Gut microflora is generally beneficial to the host [5]. It ferments the dietary fibers to produce short-chain fatty acids (SCFA) which are absorbed by the host [5]. Gut microbes also synthesize essential metabolites, such as butyrate, acetate, propionate), unsaturated and saturated medium- and long-chain fatty acids (LCFAs), and tryptophan metabolites associated with or playing crucial roles in digestion and good gut health [8]. Other than these, gut microflora provide various positive effects on the host that includes strengthening the integrity of the gut, maintaining the proper shape of the intestinal epithelium, inhibiting invasion of the pathogen, harvesting energy, and boosting the immunity of the host [5,8]. However, exposure to environmental stress, sedentary lifestyle, change in food habits, alcoholism, and over-intake/abuse of antibiotics/medicines results in the disruption of gut microbial homeostasis, leading to dysbiosis [5,6,7,8,9]. In addition, the colonization of pathogenic bacteria also leads to dysbiosis, which is linked to a number of life-threatening human diseases [5,8]. Actually, gut pathogens disrupt the protective gut mucosal layer and alleviate immune cell infiltration that subsequently leads to inflammation, tissue damage, generation of reactive oxygen species (ROS), distortion of cellular DNA, and the onset of tumor formation [5,8].

Several studies have reported that the alterations in the gut microbial homeostasis along with the pathogenic bacteria may initiate the tumorigenesis process leading to CRC [10]. Previous studies have detected considerably higher levels of *Bacteroides, Clostridium difficile, Streptococcus gallolyticus, Enterococcus faecalis, Escherichia coli, F. nucleatum,* and *Streptococcus bovis* in CRC relative to normal tissues [5,10,11]. Among these, *F. nucleatum* is considered to be one of the causative bacteria in inducing CRC in humans [10,11,12,13,14]. It is a gram-negative, anaerobic bacillus that belongs to the family *Fusobacteriaceae* [15]. This opportunist pathogen primarily resides over the human oral cavity and across the different parts of the gastrointestinal tract [10,11,12,13,14]. Initially, this bacterium was revealed as a periodontal pathogen [16]. However, the tumor and fecal samples of CRC patients exhibit a significant increase in the count of *F. nucleatum*, suggesting the contribution of this bacterium in the progression of CRC [17,18]. Further investigations refer to the lower survivability of CRC patients having an active *F. nucleatum* infection [11,14,17,18,19].

The course of CRC pathogenesis mediated by *F. nucleatum* begins with the invasion of the bacterial cells to the intestinal epithelium [17,20]. The bacterial cells are usually seen to be localized to the human colon, wherein they interact with each other and generate several virulence factors that contribute to the adherence of the bacterial cells to the colonic epithelial cells [10,17]. In this connection, the cell-surface virulent protein FadA enables the binding of *F. nucleatum* to the E-caderin protein of the colonic epithelial cells [12,17]. It triggers the activation of the Wnt/β-catenin pathway for inducing the expression of various transcription factors like lymphoid enhancer factor (LEF)/T-cell factor (TCF) for promoting the initiation and growth of the transformed colon tissue and/or malignant tumors [12,17]. In addition to this, other outer cell membrane proteins like fibroblast activation protein 2 (Fap2) and RadD facilitate the binding, colonization, and abundance of *F. nucleatum* within and across the human colonic epithelium [10,11,13,17,21].

In this context, Fap2 is considered to be the crucial bacterial protein that mediates the adhesion of bacterial cells to the human gut, specifically to the colon and rectum, to orchestrate the initiation and progression of colon carcinoma [10,11,13,17,21]. It is a 390-KDa protein comprised of 3799 amino acids encoded by the *Fap2* gene of *F. nucleatum.* It binds to the acetylgalactosamine (Gal-GalNAc) residues of the human gut epithelium resulting in the activation of multiple signaling pathways that direct the overexpression of many important cancer-critical genes, such as Annexin A1, MUC2, and STAT3, leading to the induction of tumorigenesis and metastasis of adenocarcinoma [10,13,17,21]. Intriguingly, the cell-surface expression of Fap2 has been reported to elicit the binding of *F. nucleatum* to malignant cells as well as immune cells of the host [21]. The binding of the Fap2 protein to macrophages, dendritic cells, natural killer (NK) cells, and different T-cell subsets (cytotoxic, helper, and regulatory (Treg)) around the tumor contributes to the suppression of the host immune responses that facilitate tumor growth and invasion [11,21]. In addition, this bacterial protein also participates in the physical interactions with various immunoglobulins and immunoreceptor tyrosine-based inhibitory motif (ITIM) domain receptors located on the aforementioned immune cells present in and around the gut [21]. In particular, interactions between Fap2 and the ITIM of NK cells inhibit the activity of the NK cells and promote the proliferation of colonic tumors and the progression of CRC [21]. Previous studies have shown that the attenuation of *F. nucleatum*-Fap2 prevents transmembrane signals and inhibits tumorigenesis-inducing pathways [11,17]. Thus, targeted inhibition of Fap2 may serve as a useful option to reduce the burden of *F. nucleatum* in the carcinomas of colon tissue and could emerge as an efficacious anti-tumor target in combatting CRC. In the present scenario, the lack of potential chemotherapeutics and vaccines against the early stage of CRC imposes a burden on the overall survivability of the patients. Consequently, the conceptualization of a novel vaccine candidate based on the multi-epitopes of pathogenic antigens is of utmost importance. Considering these lacunae, the present in silico study depicts the design of a multi-epitope-based peptide vaccine against the Fap2 protein of *F. nucleatum* for possible therapeutic application against human CRC.

## 2. Materials and Methods

### 2.1. Retrieval of Protein Sequences and Prediction of Linear B-Cell Epitopes

The complete sequence of the *F. nucleatum* Fap2 protein (3799 amino acids) was retrieved in FASTA format from the National Centre for Biotechnological Information (NCBI) (https://www.ncbi.nlm.nih.gov/, accessed on 14 January 2023) protein database (accession number: WP016361450.1) and was used for the study. The FBCPred (http://ailab-projects1.ist.psu.edu:8080/bcpred/predict.html, accessed on 14 January 2023) and ABCPred (http://crdd.osdd.net/raghava/abcpred/ABC_submission/, accessed on 14 January 2023) servers were explored to determine the B-cell epitopes present in the Fap2 protein and were further used to screen the common epitopes [22,23].

### 2.2. Prediction of MHC-I and MHC-II Epitopes and Determination of Their Antigenicity

The screened B-cell epitopes were used to determine the MHC-I and MHC-II epitopes using ProPred (http://crdd.osdd.net/raghava/propred/, accessed on 14 January 2023) and ProPred 1 (http://crdd.osdd.net/raghava/propred1/, accessed on 14 January 2023) servers [24]. The antigenicity of the anticipated MHC-I and MHC-II epitopes was evaluated by setting the threshold value of 0.4 using VaxiJen v2.0 server (http://www.ddg-pharmfac.net/vaxijen/VaxiJen/VaxiJen.html, accessed on 14 January 2023) [25]. During the screening process, bacteria was selected as the target organism for constructing the antigen for vaccine development.

### 2.3. Designing of Vaccine Candidates and Prediction of Secondary Structures

The MHC-I and MHC-II epitopes were linked with the linker proteins EAAAK, GPGPG, and AAY, respectively, to construct the multi-epitope antigens. In addition, an appropriate adjuvant was added at the amino-terminal end of each antigenic construct. Adjuvants were used in designing the vaccine candidates to trigger the activation of the Toll-like receptors (TLRs) like TLR1-2 dimer, TLR4–MD2 complex, TLR5, and TLR6 that are predominantly located on the surface of human immune cells. Previously, the application of TLR agonists as adjuvants was reported to induce strong T-cell and antibody-mediated responses leading to enhanced vaccine-induced long-term immune responses in the recipients [26,27,28]. We used agonists of TLR1/2 (human β-defensin protein; accession no.-AAQ09524.1), TLR4 (50S ribosomal protein of *Mycobacterium tuberculosis*; accession no.-P9WHE3.1), TLR5 (flagellin protein of *Borreliella burgdorferi* bacterial; accession no.-CAA02137.1), and TLR6 (Pam2CSK4; accession no. 3A79_C). Secondary structures of all the vaccine candidates were determined using the two feed-forward neural network methods provided in the PSIPRED 4.0 server (http://bioinf.cu.ucl.ac.uk.psipred/, accessed on 14 January 2023), and all the structures were refined following earlier reports [22,29].

### 2.4. Analyses of the Antigenicity, Allergenicity, and Physicochemical Properties of the Designed Vaccine Candidates

After obtaining the refined secondary structures of the vaccines, the VaxiJen v2.0 server was employed to evaluate the antigenicity of each vaccine candidate ensuring further validation using the AntigenPro server [30]. Next, AllergenFP v.1.0 server (http://ddg-pharmfac.net/AllergenFP/, accessed on 14 January 2023) and AllerTOP v.2.0 server (https://www.ddg-pharmfac.net/AllerTOP/, accessed on 14 January 2023) were used to test the allergenicity of the antigenic construct [31]. The physicochemical characterization and solubility of all the vaccine candidates were also determined using the in silico tools available in ExPASy-ProtParam (http://web.expasy.org/protparam/, accessed on 14 January 2023) and ProteinSol server (https://protein-sol.manchester.ac.uk/, accessed on 14 January 2023) respectively [32,33].

### 2.5. Modelling of Tertiary Structure, Refinement, and Validation

The tertiary structure of the vaccines was modeled using the I-TASSER server (https://zhanggroup.org/I-TASSER/, accessed on 14 January 2023) with subsequent refinement using the GalaxyWEB server (http://galaxy.seoklab.org/cgi-bin/submit.cgi?type=REFINE/, accessed on 14 January 2023) [34]. From the five refined 3D model structures, we selected the best structure based on the MolProbity score and the degree of stereochemical quality of each 3D structure using SAVES v6.0 server (https://saves.mbi.ucla.edu/, accessed on 14 January 2023).

### 2.6. Molecular Docking and Determination of Biophysical Interactions

The binding affinity of each vaccine construct toward the different TLRs was studied using molecular docking analysis. The 3D structures of different TLRs, viz., TLR1/TLR2, TLR4–MD2 complex, and TLR5 were retrieved from RCSB protein databank (https://www.rcsb.org/, accessed on 14 January 2023) while TLR6 was modelled following our previous reports [22,29,35]. All the structures were refined and validated before performing the molecular docking. Herein, we used ClusPro 2.0 server (https://cluspro.bu.edu/login.php, accessed on 14 January 2023) for conducting the protein–protein rigid body molecular docking experiment [36]. The docked complex comprising the vaccine peptide and TLR displaying the lowest energy-weighted score was selected as the most stable protein complex. The protein complex was further analyzed for protein–protein interactions at biophysical and molecular levels using Discovery studio 2021 Client software.

### 2.7. Normal Mode Analysis

After confirming the most stable vaccine–TLR complex, normal mode analysis was performed to analyze the conformational stability of the selected protein complex through WEBnma (http://apps.cbu.uib.no/webnma3, accessed on 14 January 2023) server [37]. This platform was exploited to study the atomic fluctuations and displacements occurring within the vaccine–TLR complexes, and critical parameters like eigenvalues, deformation plots, and correlation matrix plots were studied accordingly. Eigenvalue and frequency of deformation throughout the peptide chain were graphically represented to predict the occurrence of the rigid and non-rigid portion of the protein complexes, respectively. Moreover, the direction of the molecular motion of the two interacting proteins present in each vaccine–TLR complex was also determined using the WEBnma server.

### 2.8. Molecular Dynamic Simulation

In addition to NMA, molecular dynamics (MD) simulation trajectory analysis was performed using GROMACSv5.1 package (GROningenMAchine for Chemical Simulations) to cross-verify the structural stability of the vaccine–TLR complexes [22,35]. To construct the configuration of the docked complexes, the pdb2gmx module and force field gromos96 53a6 were used [22,35]. The system was then placed in a cubical box and solvated using a simple point charge (SPC) water model that exploited the editconf and solvate modules. The system was then neutralized by introducing a counter-ion, such as Na+ or Cl−, to achieve equilibrium. Therefore, the energy was minimized by using the steepest descent integrator for 5000 nsteps and restricting the system variables with emtol with 1000 KJ mol^−1^nm^−1^. The system was equilibrated for 5 ns before the simulation ran utilizing NVT (isothermal-isochoric) and NPT (isothermal-isobaric) ensembles. Finally, MD prediction was accomplished using timesteps of 10 ns for a total span of 100 ns. Grace 5.1.23 was used to plot the simulation data in different graphs, such as the root–mean–square fluctuation (RMSF), root–mean–square deviation (RMSD), a radius of gyration (Rg), and solvent accessible surface area (SASA) [22,35]. The conformational changes within each vaccine–TLR complex throughout the MDS process were determined by superimposing the simulated docked structures obtained from the different phases of the simulation study.

### 2.9. Codon Optimization and In Silico Cloning Preparation

Reverse translation, codon optimization, and prediction of the expression vector for in silico cloning of each designed vaccine were conducted using Java Codon Adaptation Tool (JCat) [38,39]. Codon optimization is an essential feature of cloning because it allows one to reconfigure the codons based on the host to increase the level of translational expression. The JCat server uses the amino acid sequence to produce DNA codons as well as a codon adaptation index (CAI), which indicates the unfair usage of the codon. Furthermore, the server shows the percentage of GC content and predicts translational capabilities. A GC content of 30–70% and a CAI value of 0.8 to 1 is considered optimum for adaptation within the host required for operating the translational events. We used SnapGene Software for optimization of the codon and, finally, the expression vector pET30ax was used for in silico cloning and expression of the vaccine within *E. coli* [22].

### 2.10. Determination of the Structure of the mRNA Encoding the Vaccine Peptide

The secondary mRNA structure of the vaccine was predicted by using the RNAfold webserver (http://rna.tbi.univie.ac.at//cgi-bin/RNAWebSuite/RNAfold.cgi, accessed on 14 January 2023). mRNA structure was predicted and the minimum free energy (MFE) was calculated by using a DNA or RNA sequence of the vaccine employing JCat server as depicted in our previous report [22,29,39,40,41]

### 2.11. Immune Simulation

To validate the immunological responses of the designed and in silico screened vaccines, an agent-based immune simulation technique was adopted and executed using the C-ImmSim webserver (http://www.cbs.dtu.dk/services/C-ImmSim-10.1/, accessed on 14 January 2023) [42]. The program uses the amino acid sequence (in FASTA format) of each vaccine to operate the simulation process, and the simulation mimics the administration of the vaccine to stimulate the human thymus, bone marrow, and lymph node. Moreover, it also predicts the ability of the vaccine to induce the differentiation and proliferation of different immune cells, such as cytotoxic T-cells (Tc), helper T-cells (Th), B-cells, immunoglobulins, macrophages, and cytokines, which are generally expressed in response to a vaccine. In the immune simulation experiment, five doses of the designed vaccine, 10, 20, 30, 40, and 50, were administrated through three injections at intervals of 4 weeks. The parameters 1, 91, and 181-time steps were fixed as one step, which is equal to 8 h of real life, and all other parameters were left at their default values [43].

## 3. Results

### 3.1. Presence of B-Cell, MHC-I, and MHC-II Epitopes in Fap2 Protein and Their Antigenicity

The analyses conducted with FBCPred and ABCPred servers, respectively, revealed 1747 and 399 B-cell epitopes within the amino acid sequence of Fap2. Out of these epitopes, seven common MHC-I epitopes and 3 MHC-II epitopes were screened out on the basis of their antigenicity score determined by VaxiJen v2.0 (Table 1 and Table 2). The cut-off for the VaxiJen score for selecting the MHC-I and MHC-II epitopes was set at >1.5 and >1, respectively (Table 1 and Table 2).

### 3.2. Designing the Vaccine Candidates and Prediction of the Secondary and Tertiary Structures

**Figure 1:**
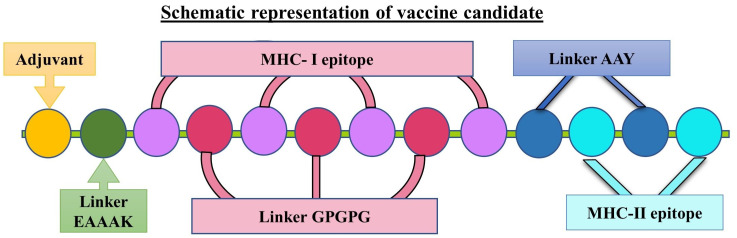
Schematic representation of the multi-epitope-based vaccine designed by linking the MHC-I and MHC-II epitopes of *F. nucleatum* with the different linkers and adjuvant.

**Figure 2:**
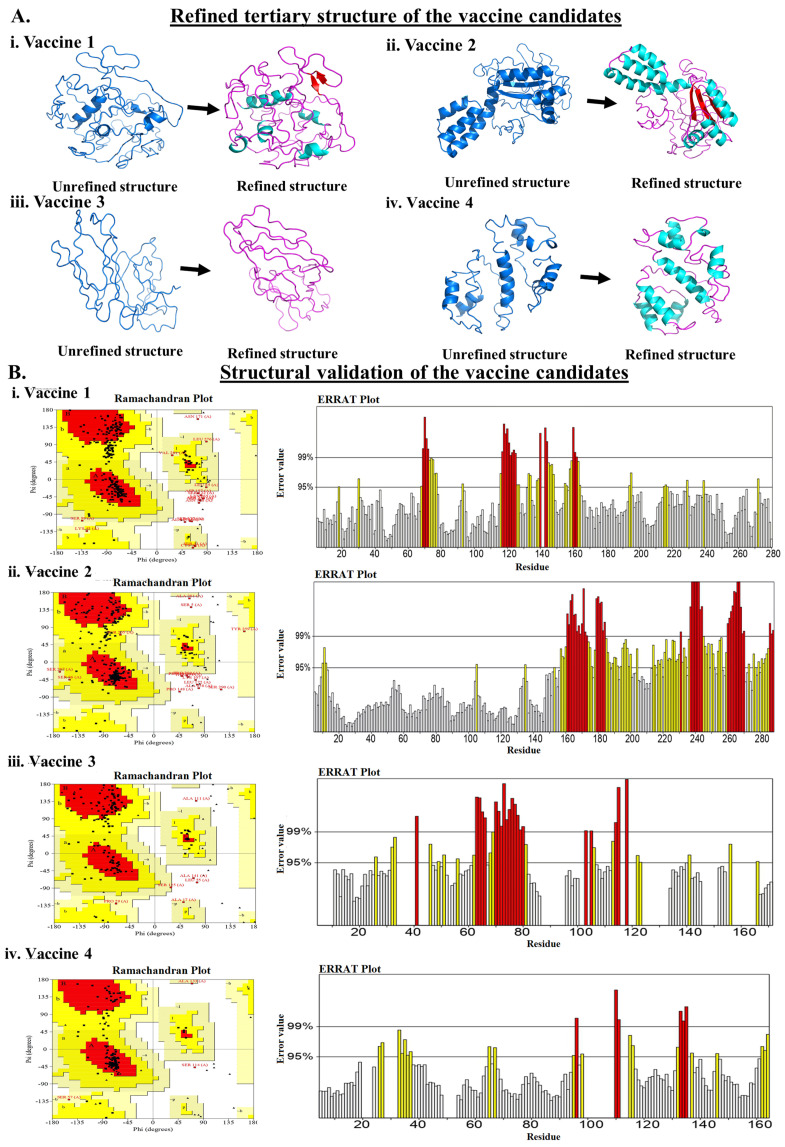
3D structure of the vaccine candidates. (**A**). Refined tertiary structure of the (**i**). VACCINE l, (**ii**). VACCINE 2, (**iii**). VACCINE 3, and (**iv**). VACCINE 4. Blue and magenta colors represent unrefined and refined structures. (**B**). Structural validation of the 3D structures. Ramachandran plot and ERRAT value plots for (**i**). VACCINE l, (**ii**). VACCINE 2, (**iii**). VACCINE 3, and (**iv**). VACCINE 4.

The vaccine construct was generated using the selected MHC-I and MHC-II epitopes, as depicted in Table 1 and Table 2. Herein, seven MHC-I epitopes were linked together using a GPGPG linker, and three MHC-II epitopes were linked with an AAY linker peptide. The various TLR agonist adjuvants were linked to the amino-terminal end of the vaccine candidate using GGGS and EAAAK linker peptides. In detail, human β defensin was linked to generate VACCINE 1, mycobacterial 50S ribosomal protein was used for VACCINE 2, *Borreliella burgdorferi* bacterial flagellin for VACCINE 3, and Pam2CSK 4 for generating VACCINE 4. The schematic diagram of the designed vaccine candidate containing 10MHC molecules, one TLR agonist adjuvant, and 10 linkers are depicted in Figure 1. The secondary structure of each vaccine construct, viz., alpha-helix, random coil, and strand region were compared graphically for all the four designed vaccine structures as given in Figure S1. The antigenicity, allergenicity, and physiochemical characterization of the different vaccine candidates were depicted in Table 3. The predicted scores for the different analyses indicate that the designed vaccines are non-allergenic, soluble, and stable stereochemical conformations. Notably, these vaccine structures were found to have a significant aliphatic index in relation to their substantial stimulatory effects in generating immunogenicity (Table 3). In addition, our study also included further refinement of the modeled 3D structures of all the generated vaccines (Figure 2A) and cross-examination of the stereochemical stability through Ramachandran plot and ERRAT analyses (Figure 2, Table 4). Ramachandran plot for each refine vaccine structure demonstrated a good stereochemical quality, wherein >90% of the constituent amino acid residues were detected in the structurally favored regions of all the four vaccines (Figure 2B, Table 4). Moreover, the ERRAT-value for the overall quality factor for the various non-bonded atomic interactions occurring within each vaccine show >50% values that resemble a high stereochemical quality in the developed 3D structures (Figure 2B, Table 4). Upon confirmation of the stereochemical quality, the refined 3D structures corresponding to the designed vaccine candidates were chosen for molecular docking study.

### 3.3. Molecular Docking of the Vaccines with Human TLRs and Exploration of the Biophysical Interactions

**Figure 3:**
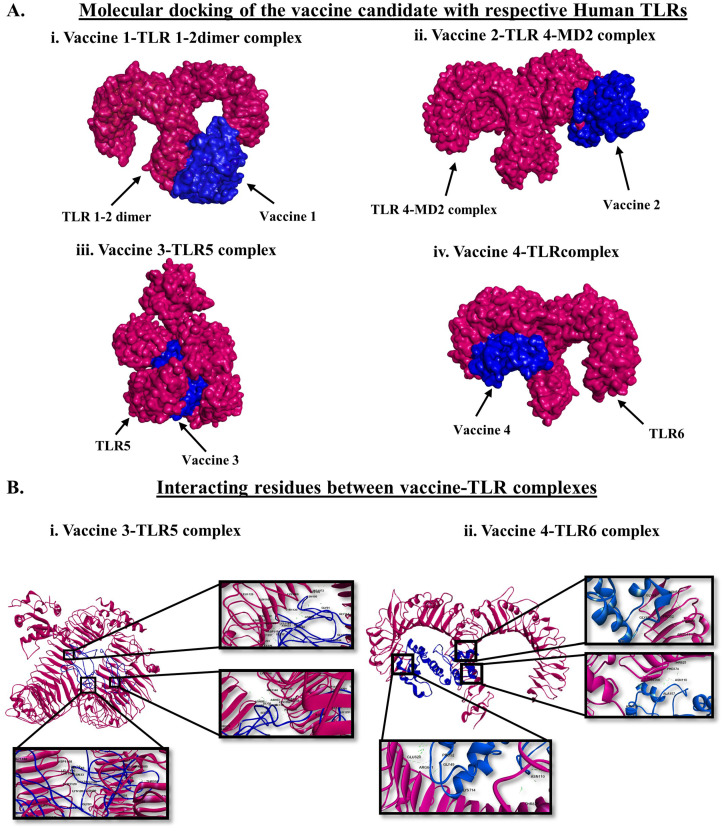
Molecular docking and biomolecular interaction between the vaccine candidates and human TLRs. (**A**). Molecular docking between (**i**). VACCINE l with TLR1/2 dimer, (**ii**). VACCINE 2 and TLR4–MD2 complex, (**iii**). VACCINE 3 and TLR5, and (**iv**). VACCINE 4 with TLR6. (**B**). Interacting residues participating in the molecular docking between (**i**). VACCINE 3 and TLR5, and (**ii**). VACCINE 4 with TLR6.

Vaccines are designed to induce immune sensitization against a specific pathogen or group of pathogens. We examined the immunostimulatory activity of the designed vaccines by studying the efficiency of the vaccine candidates to interact with the different human TLRs. TLRs are the primary innate immune sensors that selectively recognize pathogen-associated molecular patterns (PAMPs) and shape both innate and adaptive immune responses [28,44]. Herein, molecular docking revealed the differential interactions of the vaccine candidates with the human cell surface TLRs (Table 4 and Figure 3A). We observed significant interactions between VACCINE 1 and TLR1/TLR2 dimer, VACCINE 2 and TLR4–MD2 complex, VACCINE 3 and TLR5, and VACCINE 4 and TLR6 (Table 4 and Figure 3A). Considering all four different vaccine–TLR complexes, two protein complexes comprising VACCINE 3–TLR5 and VACCINE 4–TLR6 were found to display the most negative binding energy score (Table 4 and Figure 3B). These protein complexes were further selected for studying the biomolecular interactions and biophysical stability. As depicted in Table 5, the VACCINE 3–TLR5 complex exhibits 57 hydrogen bonds and nine hydrophobic interactions while the VACCINE 4–TLR6 complex revealed 25 hydrogen bonds and four hydrophobic interactions (Table 5). Therefore, the higher abundance of non-covalent interactions between the aforementioned vaccines and TLRs is most likely to play a key role in stabilizing the physical protein–protein interactions between the interacting partners.

### 3.4. Normal Mode Analysis (NMA)

**Figure 4:**
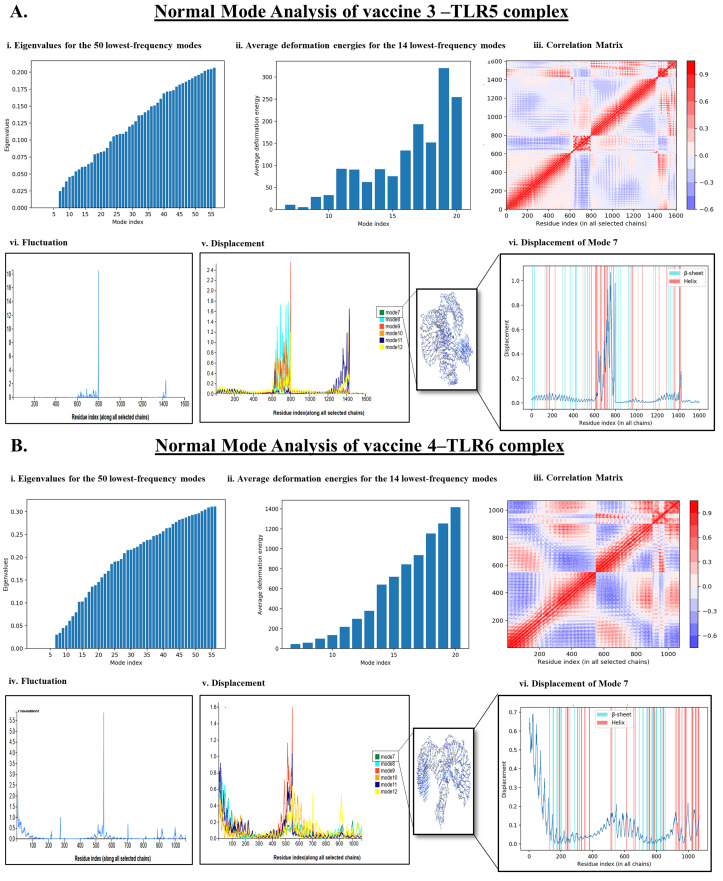
Normal mode analysis depicting the stability and flexibility of the docked structures (**A**). NMA of VACCINE 3–TLR 5 complex depicting (**i**). Eigenvalues, (**ii**). Average deformation energies of different modes, (**iii**). correlation matrix, (**iv**). Fluctuation plot, (**v**). Displacement plot, and (**vi**). Displacement of the best mode (mode 7). (**B**). NMA of VACCINE 4–TLR-6 complex representing the different plots for (**i**). Eigenvalues, (**ii**). Average deformation energies of different modes, (**iii**). Correlation matrix, (**iv**). Fluctuation plot, (**v**). Displacement plot, and (**vi**). Displacement of the best mode (mode 7).

NMA is a useful molecular simulation technique to evaluate the binding stability of two interacting proteins occurring as a complex across different modes throughout the structure of the protein complex [45]. Herein, our study revealed that VACCINE 3-TLR5 and VACCINE 4–TLR6 complexes possess a significant degree of biophysical and perturbational stability throughout the simulation phase (Figure 4). Studies employing WEBnma demonstrated the direction of the domain mobility of the vaccine peptide and human TLR toward each other within the complex, as presented by the arrows in Figure 4. Occurrences of molecular and atomistic motion in the course of protein–protein interactions were evaluated by the correlation matrix that resembles the relationship amongst the pairs of residues in each vaccine–TLR complex (Figure 4A(iii),B(iii)). As given in Figure 4A(iii),B(iii), red, blue, and white colors, respectively, indicate the occurrence of correlated, anti-correlated, and uncorrelated pairs of residual motion with the protein complexes. The high degree of stability of the VACCINE 3–TLR 5 and VACCINE 4–TLR6 complexes was also deciphered by a low eigenvalue of 0.025 and 0.05, respectively (Figure 4A(i),B(i)). The energy associated with each mode was reflected in deformation energies and eigenvalues, which are inversely proportionate to the amplitude of the motion given by the associated modes (Figure 4A(ii),B(ii)). The atomic displacements of the six lowest-frequency modes are shown in Figure 4A(v),B(v). The displacement of mode 7 is shown in Figure 4A(vi),B(vi). The fluctuations of the atomic position are shown in Figure 4A(iv),B(iv). This evidence of deformability and fluctuations within the protein complex during the course of simulation collectively revealed a high degree of structural flexibility and stability of the two protein complexes formed between the designed vaccines and human TLRs.

### 3.5. Molecular Dynamics Simulation (MDS) Trajectory Analysis

**Figure 5:**
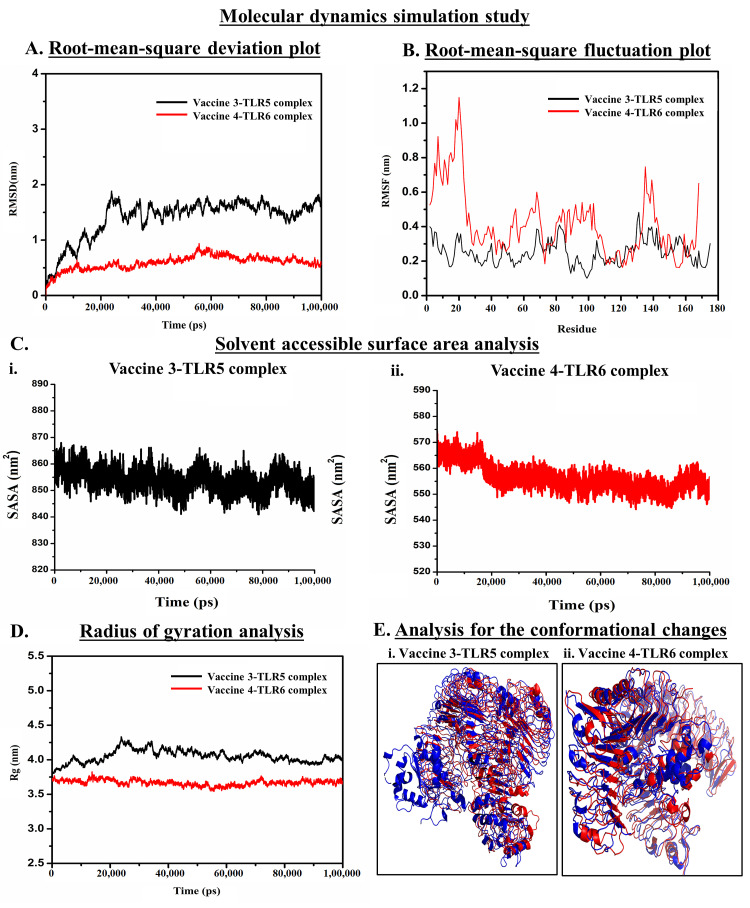
MDS trajectory analysis showing the biophysical stability of VACCINE 3–TLR5 and VACCINE 4–TLR6 complexes. (**A**). RMSD plot generated across the simulation period shows the deviation in the stability of the vaccine–TLR complexes. (**B**). RMSF plots generated by combining the residual fluctuation within the two vaccine–TLR complexes during the entire simulation phase. In the RMSD and RMSF plots, VACCINE 3–TLR5 and VACCINE 4–TLR6 complexes are shown in black and red color, respectively. (**C**). Graphical demonstration of SASA for (**i**). VACCINE 3–TLR5 and (**ii**). VACCINE 4–TLR6 complexes (**D**). Rg plots showing the movement of the interacting proteins within the vaccine–TLR complexes. Black and red lines represent the VACCINE 3–TLR5 and VACCINE 4–TLR6 complexes, respectively. (**E**). Analysis of the conformational changes by the superimposing structure of (**i**). VACCINE 3–TLR5 unbounded state in red color and binding after 100 ns in blue color, and (**ii**). VACCINE 4–TLR6 unbounded state in red color and binding after 100 ns in blue color.

Findings of NMA regarding the stability of the vaccine–TLR complexes were re-examined and verified using MDS trajectory analyses (Figure 5). MDS employs different force fields and examines the effects of solvents to explore the physical interactions between the two proteins occurring as a complex [22,29]. In this study, the biophysical stability of the VACCINE–3-TLR5 and VACCINE–4-TLR6 complexes was analyzed by assessing the RMSD, RMSF, Rg, and SASA plots within the definite MD trajectory (Figure 5).

#### 3.5.1. Analysis of the Stability of Vaccine–TLR Complexes Using RMSD

RMSD plot demonstrates the conformational stability of the protein complexes containing the designed VACCINE (3 and 4) and human TLR (5 and 6) complexes (Figure 5A). In the RMSD plot, the black and red bands, respectively, indicate the changes or fluctuation in the conformation of the VACCINE 3–TLR5 and VACCINE 4–TLR6 complexes with time, respectively, up to 100 ns. After an initial rapid change in the RMSD until 25 ns, the VACCINE 3–TLR5 complex was found to achieve stability with a very minute degree of fluctuation. However, the VACCINE 4–TLR6 complex achieved its stability since the beginning of the MDS process. The value of the average RMSD of the stable conformations of the VACCINE 4–TLR6 complex also resembles a high degree of conformational stability of the protein complex.

#### 3.5.2. Analysis of Residual Flexibility

RMSF was employed to demonstrate the flexibility of the two vaccine–TLR complexes for each amino acid residue and the role of each residue in accounting for the mean flexibility of the whole protein occurring as a complex. RMSF is a popular choice to understand the dynamicity of protein–protein interaction [22,29]. The overall flexibility of the designed vaccine candidates determined by the RMSF plot suggests that both the vaccine candidates possess most of the amino acid residues within a very low RMSF range. This result infers that the docking of vaccine candidates with the respective TLRs exhibits a high degree of flexibility which could be correlated with their efficiency in augmenting TLR responses (Figure 5B).

#### 3.5.3. Analysis of Solvent-Accessible Surface Area

SASA plots were studied to determine the area of each vaccine–TLR complex accessible to solvent. As depicted in Figure 5C, the average quantitative value of SASA for the VACCINE 4–TLR6 protein complex displayed a minimum degree of fluctuation that indicates toward a high stability of the complex formed between VACCINE 4–TLR6.

#### 3.5.4. Analysis of Compactness

Rg is the measure of the root–mean–square distance of a collection of residues from their center of mass. Our study indicates that the VACCINE 4–TLR6 complex exhibits an average Rg value of 3.83 nm, which designates a lower fluctuation and compactness over the generation of simulation time (Figure 5D). It also suggests that the docked complex possesses strong interactions between its components, i.e., the vaccine and TLR6, which is expected to contribute to the greater compactness of the complex formed by the two proteins.

#### 3.5.5. Determining the Conformational Changes

Changes in the native conformation of the interacting proteins during a protein–protein interaction and after the formation of a complex are considered to be an important criterion in forming a stable complex [22,29]. The conformational changes in the conformation of the vaccine and TLR across the trajectory of the MDS were recorded through the superimposition of the native unbound structure of the vaccine and TLRs with the vaccine-bounded structures over the different time steps. Herein, the comparison of the bound state of VACCINE 3–TLR5 and VACCINE 4–TLR6 complexes with the individual unbound states of the two interacting partners revealed a significant degree of conformational changes in both the proteins that were found to persist in the entire MDS course of 100 ns (Figure 5E).

### 3.6. Codon Optimization and In Silico Cloning for the Production of Recombinant Protein

**Figure 6:**
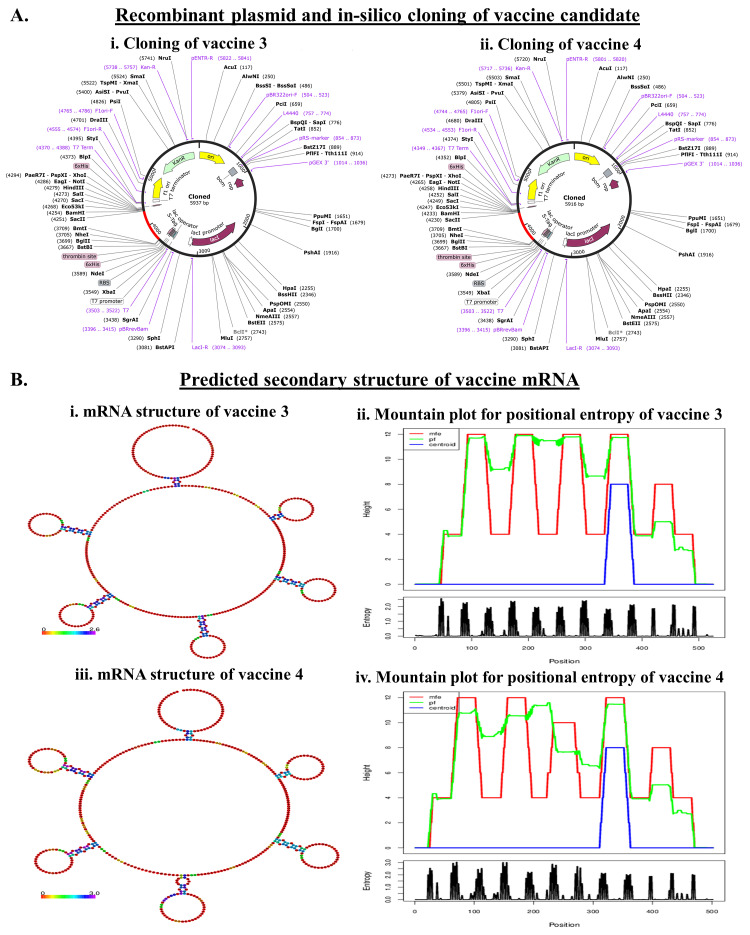
In silico cloning of the gene sequence of the vaccines in pET30ax vector and the predicted secondary mRNA structures. (**A**). Recombinant plasmid and in silico cloning of (**i**). VACCINE 3, (**ii**). VACCINE 4. The vaccine region is represented by red color. (**B**). Predicted secondary structure of vaccine mRNA (**i**). Minimum free energy (MFE) structure and (**ii**). Mountain plot exhibiting the comparative relation between the MFE, the thermodynamic ensemble, and the centroid structure of the mRNA of VACCINE 3. (**iii**). Minimum free energy (MFE) structure and (**iv**). Mountain plot exhibiting the comparative relation between the MFE, the thermodynamic ensemble, and the centroid structure of the mRNA of the VACCINE 4. Positional entropies are also reflected in the plot where the plateaus and slopes respectively dignify the loops and helix.

Any peptide vaccine needs proper cloning in a suitable vector for further in vivo validation. We separately cloned the two vaccine constructs. The reverse translation and codon optimization of VACCINE 3 and VACCINE 4 peptides, respectively, show 526bp and 508bp nucleotide sequences. The CAI values of the optimized sequences of VACCINE 3 were 0.1584 and the GC content was 57.69 while VACCINE 4 showed a CAI value of 0.1924 and the GC content was 57.91, indicating a high possibility of expressing the recombinant vaccines within *E. coli*. The recombinant plasmid vector pET30ax contains a T7 promoter, a lac operator, a translation initiation region (TIR), a conserved AGGAGG sequence (Shine–Dalgarno sequence), and a nucleotide sequence corresponding to poly-linkers or multiple cloning sites. The vaccine cDNA was incorporated within the vector after the in silico digestion of the vector with EcoRI using SnapGene software (Figure 6A).

### 3.7. mRNA Structure Prediction and Durability Analysis of the Designed Vaccine

The mRNA structures of the designed vaccines derived using RNAfold and JCat collectively exhibited a centroid secondary structure conformation with MFE (Figure 6B). Notably, the negative free energy for VACCINE 3 (−24.49 kcal/mol) and VACCINE 4 (−21.39 kcal/mol) confirm that the in vivo mRNA form of the vaccines is stable and durable (Table S1).

### 3.8. Immune Simulation

**Figure 7:**
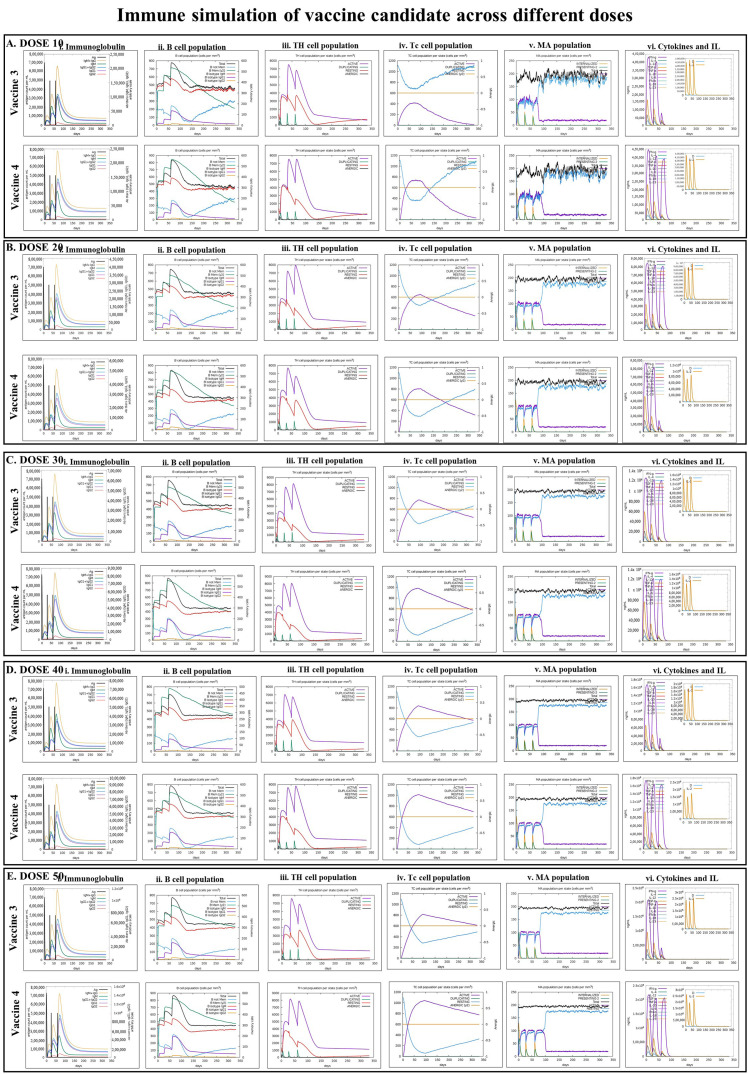
In silico immune simulation showing the comparative efficacy of the designed vaccines in inducing immune responses. Immune response for the simulation Volumes of (**A**) dose 10, (**B**), dose 20, (**C**), dose 30, (**D**), dose 40, and (**E**), dose 5 showing the (**i**). production of several subclasses of immunoglobulin (colored lines) in response to vaccine injection (black vertical lines), (**ii**). B-cell richness after three injections, (**iii**). Evolution of Th-cells after the injections, (iv). Evolution of Tc-cells after the injections. (**v**). Production of macrophage. (**vi**). Production of cytokine and interleukin (IL).

After confirming the stability of the two vaccine constructs, i.e., VACCINE 3 and 4 in binding human TLR5 and 6, respectively, their efficiency in inducing adaptive immunity was examined by immune simulation. The results of the immune simulation by the C-Immsim server revealed that the first two injections of the developed vaccine generate a low response of immunoglobulin IgM, IgG1, and IgG2, but the third injection empowers a high degree of immunoglobulin response as the levels of IgM+IgG and IgG1+IgG2 immunoglobulin were found to be elevated (Figure 7(i)). Comparing the immune responses generated separately in response to the two different vaccines demonstrated that the antigenicity of VACCINE 4 is higher to that of VACCINE 3 (Figure 7). In comparison to the other immunoglobulins, such as IgM, IgG1, and IgG1+IgG2, the abundance of IgG+IgM states that VACCINE 4 elicits a better immune response (Figure 7(i)). In addition to this, the proportion of B-cells, Tc-cells, Th-cells, macrophages (MA), and cytokine production were also elevated in response to VACCINE 4, which indicates toward the future promise of VACCINE 4 in shaping immunity against *F. nucleatum* (Figure 7). Our simulation data also predicted the dose of VACCINE 4 for inducing an effective and durable immune response. It was found that a minimum simulation volume of 10 units of VACCINE 4 is efficient enough to produce a significant level of an immune response against *F. nucleatum* (Figure 7A).

## 4. Discussion

Changes in lifestyle, food habits, and perturbation of gut microflora leading to the disruption of gut homeostasis are the major contributing factors behind several human diseases, such as irritable bowel syndrome (IBS), inflammatory bowel disease (IBD), ulcerative colitis (UC), and Crohn’s disease, as well as life-threatening gut cancers [4,5]. Under normal circumstances, the gut microflora suitably regulate the physiological and immune homeostasis of the gut, and such a healthy gut efficiently maintains the physiological homeostasis of the human body through gut–organ axes [4,5]. In fact, the ratio of *Firmicutes* and *Bacteroides*, commonly known as the F/B ratio, is considered to be a major influential determinant in establishing and maintaining a healthy human gut [4,5]. As stated earlier, gut microbiota is vulnerable to various consequences that perturb normal gut health, and such a change in the gut microbial consortium is known as dysbiosis [4,5]. This dysbiosis triggers the induction of inflammatory responses, damage in the colonic protective mucosa, immigration of pathogenic bacteria, and expression of their antigenic genes [4,5]. All these subsequently lead to the transformation of normal colon tissue into carcinomas. The increase in the population of *F. nucleatum* within the colon tissue alters the properties of the colon cells and leads to the overactivation of immune responses and alteration in the level of different immunoglobulins [46]. As described in the introduction section, the Fap2 protein of *F. nucleatum* is an essential mediator in the adhesion of the bacterium with the colonic epithelial cells through the bacterial outer membrane and results in the oncogenesis and pathogenesis of CRC [10,11,12,13,14]. Previous studies have suggested Fap2 as a potential target for the conception of anti-cancer therapy [10,11,12,13,14,17,21]. To date, a number of strategies have been implicated in manipulating the genetic programming of Fap2 through inducing mutations, applying genetic engineering, and employing RNA-mediated interference strategies (siRNAs and shRNAs) [11,13,17,21]. Moreover, the targeted induction of tumor suppressor genes and immune regulatory peptides have also been administered for counteracting the Fap2 protein of *F. nucleatum* [11,13,17,21]. However, a vaccine candidate for the targeted inhibition of *F. nucleatum*-induced CRC is not available to date. Thus, the use of Fap2 as an antigenic determinant for the development of a multi-epitope-based peptide vaccine may trigger both cell-mediated and humoral immunity. In this regard, our study exploits the Fap2 protein of *F. nucleatum* to develop a novel and efficacious vaccine construct against CRC.

Herein, we adopted a reverse vaccinology approach for designing the vaccine, as this methodology has been considered a popular choice for developing effective vaccines over the years [22]. In order to construct the vaccine candidate, the antigenic epitopes present in the bacterial protein Fap2 were retrieved. Both B-cell and T-cell epitopes within an antigen are critical in shaping the humoral and cytotoxic immune responses. B-cell epitopes upon binding activate the B-cells to stimulate the production and release of the antibodies to neutralize the antigens and produce memory B-cells [22,29]. However, the MHC-II epitopes bind with the CD4^+^T-cells and activate the helper T-cells to release cytokines that in turn activate B-cells and CD8^+^T-cells for the cell-mediated immune responses [22,29].

The present study began with the retrieval of the protein sequence of Fap2 obtained from NCBI which was used to predict the various B and T-cell epitopes, which were further refined to acquire a total of 10 B-cell epitopes, out of which, seven were found to bind MHC-I while three epitopes were found binding MHC-II (Table 1 and Table 2). After confirming the epitopes, antigenicity as well as other immunological properties (allergenic nature, hydrophobicity, etc.) were analyzed. In reverse vaccinology-based vaccine design, epitope screening prior to constructing a vaccine is considered advantageous in generating intense and specific immune responses, including antibody response [22,29]. Moreover, this strategy restricts the unwanted participation of the non/less allergenic peptides and amplifies the immunogenicity of the actual antigen as well as the binding of the vaccine peptide to its desired target [16,22,29]. After obtaining the preliminary design of the vaccine, TLR-specific adjuvants such as human β-defensin protein, mycobacterium 50S ribosomal protein, flagellin protein of *Borreliella burgdorferi*, and Pam2CSK4 were also added separately at the N-terminal end of the vaccine peptides to increase the specificity and antigenicity of the same (Figure 1). In this direction, earlier researchers have documented the induction of strong antibody-mediated and T-cell responses after the administration of TLR agonists in the form of an adjuvant [22,26,27,29]. TLR signaling pathways play multiple important roles in regulating and maintaining the intensity and durability of proinflammatory responses across human colon tissues [47]. In addition, a number of TLR agonists are now considered a popular choice in preparing vaccine formulations for clinical applications to combat various infectious as well as inflammatory diseases [28,44]. In our study, the joining of the epitopes by applying appropriate linkers along with the addition of immunostimulatory adjuvants resulted in the formation of four different vaccine constructs, namely, VACCINE 1, VACCINE 2, VACCINE 3, and VACCINE 4 (Figure 1). In order to characterize the vaccines, the secondary structural components as well as other physico–biochemical and immunobiological properties were determined, and these observations indicated the designed multi-epitope vaccines as stable proteins (Table 3). These observations were re-verified through analyzing the tertiary configuration of the individual vaccine structure following stereochemical refinement (Figure 2). Our observation on the Ramachandran plots demonstrated a good stereochemical fitness of the vaccine proteins (Figure 2, Table 4). Since we aimed to make a vaccine that could target the human TLRs for eliciting an intense immune response, we performed molecular docking by using the 3D structures of the designed antigenic constructs and human TLRs. However, the four different vaccine designs displayed a different degree of binding to the target TLRs. Inside the host body, the binding of a ligand to the different TLRs activates the inflammatory signaling cascade and results in the release of cytokines, chemokines, and type 3 interferon (IFN-γ) [28]. These inflammatory mediators are essential for the activation and expansion of various innate (macrophages, NK cells, dendritic cells) and adaptive (B- and T-cells) immune cells [28]. Thus, we explored the binding of the vaccine candidates with the cell surface TLRs, and the most significant binding was observed for the VACCINE 3–TLR5 and VACCINE 4–TLR6 complexes (Figure 3). Moreover, such bindings were found to be mediated by the formation of hydrogen bonds, hydrophobic interactions and electrostatic interactions (Figure 3, Table 5). Based on the affinity of the antigens in binding the TLRs, two vaccines (VACCINE 3, and VACCINE 4) were selected for MDS trajectory study and immune simulation (Figure 4, Figure 5 and Figure 6).

NMA and MDS collectively evidenced a high degree of biophysical stability and stable binding dynamics in the course of protein–protein interactions occurring in each TLR–vaccine complex (Figure 4 and Figure 5). In NMA, several modes of the docked structures were generated to predict the stability based on the eigenvalue, deformation energy, displacement and fluctuation plot, and correlation matrix [37]. Our study indicates the best result for the VACCINE 3 and VACCINE 4 construct, which resemble a high degree of deformation contributing to the flexibility as well as stability in the protein complexes formed by the vaccine peptide and TLRs (Figure 4). VACCINE 3 and 4 were further selected for the MDS study. The simulation study reveals that the RMSD, RMSF, Rg, and SASA plots for the two different vaccine–TLR complexes show sustained stability across the entire phase of the simulation (Figure 5). However, the comparative evaluation of the binding between the vaccines and the TLRs reveals that the VACCINE 4–TLR6 complex is much more stable, and this complex represents the highest affinity for the vaccine toward the TLR (Figure 5). Considering this, VACCINE 4 was selected as the most efficacious vaccine. Notably, this multi-epitope vaccine was found to occupy the extracellular domain of the target TLRs, and the binding resulted in a significant degree of conformational changes that are most likely to induce signaling cascade for NF-κB activation and the subsequent release of the cytokines. Being the most efficacious, VACCINE 4 was subjected to in silico molecular cloning in pET30ax vector to produce the recombinant form of the vaccine in *E. coli* (Figure 6A). Interestingly, the secondary mRNA structure of the VACCINE 4 also indicated a high degree of stability and durability in in vivo conditions (Figure 6B). Finally, our immune simulation data clearly revealed that VACCINE 4 has enough potential to elicit vaccine-mediated immune responses under simulated conditions (Figure 7). We observed the sensitization of the cells’ primary lymphoid organs (bone marrow) and secondary lymphoid organs (thymus and lymph node) to induce the differentiation of various immune cells, including antibody-producing and memory B-cells as well as various T-cell subsets to enhance the levels of serum immunoglobulins and different cytokines/chemokines (Figure 7). Since all the vaccines are meant to induce the level of circulating antibodies, our projected VACCINE 4 was found to be extremely potential to increase the level of IgG1 + IgG2, IgM, and IgG + IgM during primary, secondary, and tertiary immune responses under simulated conditions (Figure 7A). Most importantly, the designed peptide vaccine was found to be non-allergenic. Collectively, all these pieces of evidence collectively signify VACCINE 4 as a novel promising therapeutic for intervening in *F. nucleatum*-induced human CRC. The most intriguing feature about our designed vaccine peptide is that these vaccines are effective in inducing both adaptive and innate responses under simulated conditions. Since all the experimental findings were obtained in silico, we do welcome the participation of the scientific community for experimental validation which may gift mankind a new weapon to fight against CRC in near future. Notably, several earlier studies have documented the utility in designing efficient vaccines which were indeed found efficacious when tested through experimental immunization and/or pre-clinical trials [48,49,50]. For example, the in silico multi-epitope vaccine candidate 4CMenB designed by combining the three antigens of *Neisseria meningitidis*, namely, Neisserial Heparin Binding Antigen (NHBA), Factor H binding protein (fHbp), and Neisseria Adhesin A (NadA), was found to elicit strong bactericidal immune response in the recipients of a different age group [50]. More interestingly, this vaccine was approved for immunization in 2013 and 2015, respectively, in Europe and the United Kingdom [49]. In the recent past, another immunoinformatic-based multi-epitope vaccine, Ov-DKR-2, designed to combat human filariasis, was found to cross react with the sera of individuals suffering from onchocerciasis as well as loiasis [48]. In addition to these, our earlier report on the design of a novel multiepitope-based universal vaccine, “AbhiSCoVac”, having the potential to target all the different strains of COVID-19 using bioinformatics and immune-informatics studies has been widely accepted by the scientific community worldwide and currently is undergoing pre-clinical testing in an animal model [51]. Taken together, we hope that the present vaccine construct will also be useful in inducing adequate immune response against *F. nucleatum* infection to reduce the risk of CRC.

## 5. Conclusions

CRC is one of the deadliest and most common forms of gut cancer worldwide. The changes in the gut microenvironment following the induction of inflammatory and immunogenic alterations of gut homeostasis drive tumorigenesis and metastasis. The role of *F. nucleatum* in the pathogenesis of CRC is well established and thus therapeutic amelioration of the growth and metabolism of *F. nucleatum* can provide new strategies of intervention. Targeting the Fap2 protein inhibits the adherence to the colonic epithelium and restricts the pathogenesis of *F. nucleatum* in the course of the pathogenesis of CRC. Thus, this study was aimed to develop a novel efficacious vaccine candidate to generate a strong immune response against the *F. nucleatum* Fap2 protein to prevent the onset of *F. nucleatum*-induced CRC in humans. Our study formulates a multi-epitope-based peptide vaccine using both T-cell and B-cell epitopes occurring in the Fap2 protein that efficiently target the human TLRs to elicit an intense humoral and cell-mediated immune response. Our in silico study designed four different vaccine candidates, out of which Pam2CSK4 adjuvant coupled VACCINE 4 was found to be the most efficient in binding human TLR6. Moreover, this vaccine generates extremely intense and durable immunoglobulin-mediated as well as T-cell-mediated responses. Therefore, it is expected that this novel vaccine could be effective in inducing a protective immune response in recipients who have an active *F. nucleatum* infection or have the risk of *F. nucleatum* infection. The limitation of this study is the application of in silico-based computational methods for the experimental results. Although the in silico methods used in this current study are highly authentic and validated, it is very much necessary to validate our results using an experimental design in both in vivo and in vitro methods before mass administration to humankind. Our study welcomes experimental validation by the experimental biologists to confirm all results experimentally over in vivo and in vitro experiments. The allergenicity and antigenicity of the vaccine candidates are required to be examined in an animal model before administration. Thus, this study opens up new areas of research for the development of a vaccine against CRC.

## Figures and Tables

**Table 1:** Selected MHC-I epitopes of Fap2 based on VaxiJen Score > 1.5.

Sl. No.	B-Cell Epitopes	MHC-I Allele	MHC-I Epitopes	VaxiJen Score
1.	NADGSNNTTMTNMVNK	HLA-A1	NADGSNNTT	2.3648
2.	NININGDSSIGVGLLQ	HLA-A1	NGDSSIGVG	1.7041
3.	NININGDSSIGVGLLQ	HLA-A-0205	GDSSIGVGL	1.6222
4.	ASKATNDSNGTITLDT	HLA-A1	TNDSNGTIT	1.6262
		HLA-A-0205	KATNDSNGT	2.2960
5.	SVGIFAKNNGTNDTAK	HLA-A1	NNGTNDTAK	1.6448
		HLA-A-0205	KNNGTNDTA	1.5250
6.	VGTITLKNSTVSNGSS	HLA-A1	NSTVSNGSS	1.7004
7.	PASPDPNKLEIETTSN	HLA-A1	KLEIETTSN	1.5014
		HLA-A-0205		

**Table 2:** Selected MHC-II epitopes of Fap2 based on VaxiJen Score > 1.

Sl. No.	B-Cell Epitopes Sequence	MHC-II Allele	MHC-II Epitopes	VaxiJen Score
1.	NININGDSSIGVGLLQ	HLA-DRB1_0301	INGDSSIGV	1.3077
2.	SGTIIMKNQNSVGILG	HLA-DRB1_0101 HLA-DRB1_0102	MKNQNSVGI	1.1398
3.	VGTITLKNSTVSNGSS	HLA-DRB1_0301	LKNSTVSNG	1.0256

**Table 3:** In silico assessment of antigenecity, allergenicity, solubility, and physicochemical properties of the four designed vaccine candidates.

Characteristics	VACCINE 1	VACCINE 2	VACCINE 3	VACCINE 4
VaxiJen Score	1.308	0.9979	1.3648	1.4968
Antigen-Pro Score	0.81	0.87	0.76	0.75
Solubility	0.664	0.85	0.731	0.728
No. of amino acids	284	292	175	168
Molecular weight (in Dalton)	28,348.97	28,388.16	16,402.45	15,668.6
Theoretical Isoelectric point (pI)	5.58	4.54	4.62	6.37
Formula	C_1190_H_1906_N_358_O_425_S_10_	C_1215_H_1963_N_341_O_434_S_3_	C_673_H_1079_N_209_O_261_S_4_	C_644_H_1032_N_202_O_250_S_2_
Total no. of atoms	3889	3956	2226	2130
Estimated half-life	30 h (mammalian reticulocytes, in vitro). >20 h (yeast, in vivo). >10 h (*Escherichia coli*, in vivo)	30 h (mammalian reticulocytes, in vitro) >20 h (yeast, in vivo). >10 h (*Escherichia coli*, in vivo)	30 h (mammalian reticulocytes, in vitro). >20 h (yeast, in vivo). >10 h (*Escherichia coli*, in vivo)	1.2 h (mammalian reticulocytes, in vitro) >20 h (yeast, in vivo) >10 h (*Escherichia coli*, in vivo)
Instability index	25.11	10.71	5.87	3.51
Aliphatic index	57.01	69.97	46.34	41.31
Grand Avg. of hydropathicity (GRAVY)	−0.621	−0.339	−0.655	−0.812

**Table 4:** The ramachandran plot, ERRAT values, and Docking Scores with Human TLRs of respective vaccines.

Name of Vaccine	ERRAT Value	Percentage of Protein Residue in the Favored Region (Ramachandran Plot)	Docking Score
VACCINE 1	0.81	91.8%	−693.1
VACCINE 2	0.87	95.1%	−624.4
VACCINE 3	0.76	95.8%	−1096.6
VACCINE 4	0.75	97.3%	−853.8

**Table 5:** Interactions between the Vaccine candidate and the targeted TLRs.

Vaccine Name	Targeted TLR	Hydrogen Bond	Hydrophobic Interaction	Electrostatic Bond
Vaccine 3	TLR 5	57	9	8
Vaccine 4	TLR 6	25	4	2

## Data Availability

All data generated or analyzed during this study are included in this article.

## Supplementary Materials

The following supporting information can be downloaded at: https://www.mdpi.com/article/10.3390/vaccines11030525/s1, Table S1: Analysis of thermodynamic ensemble prediction of secondary mRNA structures of vaccine candidates; Figure S1: Secondary structure of the multi-epitope-based vaccines showing coiled, helix, and strand regions. i. VACCINE-l, ii. VACCINE-2, iii. VACCINE-3, and iv. VACCINE-4.
