# Supplementary material for: Cell Surface Fibroblast Activation Protein-2 (Fap2) of Fusobacterium nucleatum as a Vaccine Candidate for Therapeutic Intervention of Human Colorectal Cancer: An Immunoinformatics Approach
Source: Vaccines (Basel). 2023 Feb 23;11(3):525. doi: 10.3390/vaccines11030525 (PMC10056511; doi:10.3390/vaccines11030525)

Supplementary Material

Table S1. Analysis of thermodynamic ensemble prediction of secondary mRNA structures of vaccine candidates.

mRNA Structure characterization	Vaccine 3	Vaccine 4
Free energy	-24.49 kcal/mol	-21.39 kcal/mol
Frequency of MFE Structure	0.99%	0.14%
Ensemble Diversity	55.13	51.91

Figure S1. Secondary structure of the multi-epitope-based vaccines showing coiled, helix, and strand regions. i. VACCINE-l, ii. VACCINE-2, iii. VACCINE-3, and iv. VACCINE-4.

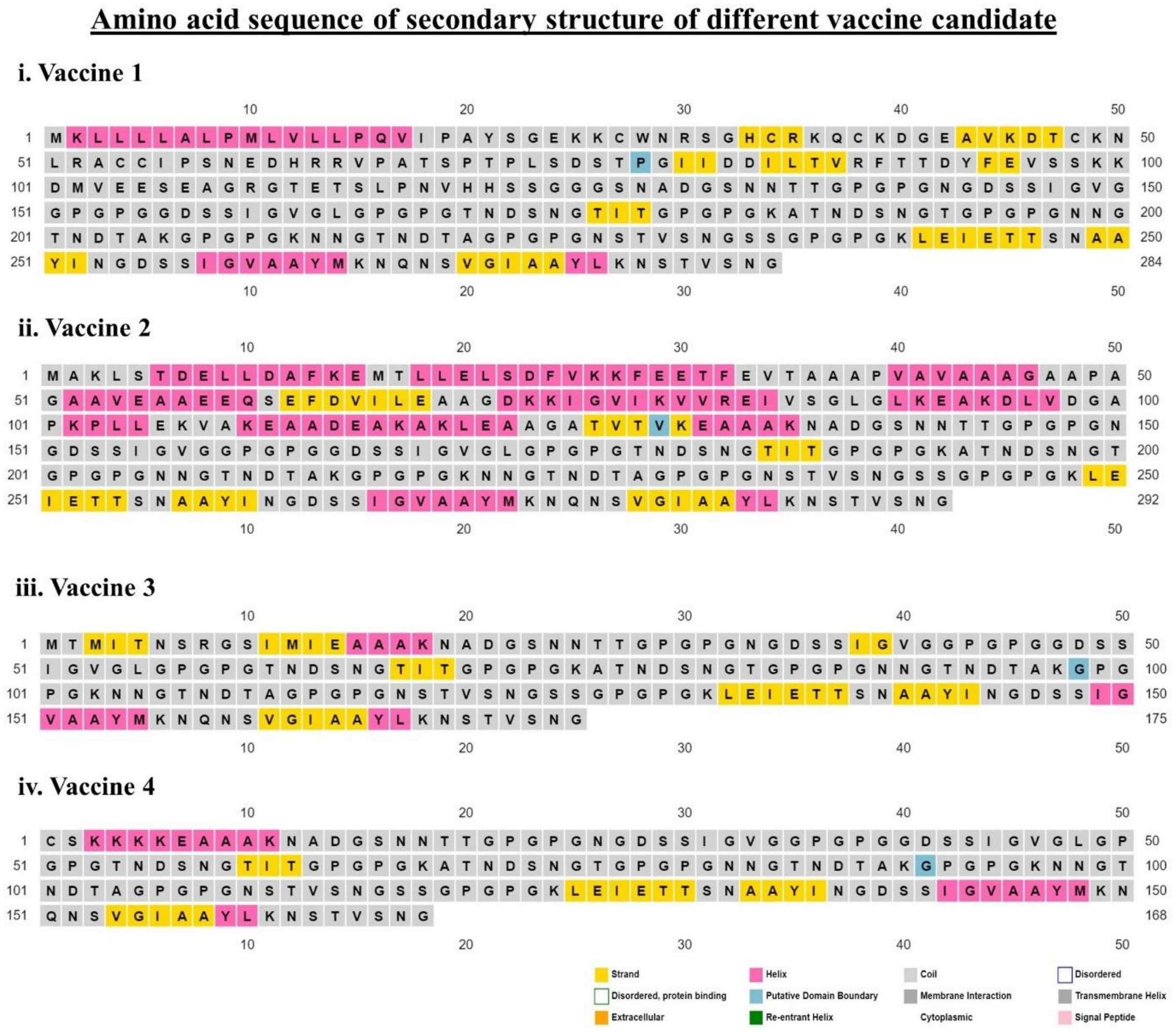

Supplement: Supplementary file 1 [file vaccines-11-00525-s001.zip › vaccines-2193110-supplementary.pdf]
